# *In vivo* singlet state filtered nuclear magnetic resonance: towards monitoring toxic responses inside living organisms†

**DOI:** 10.1039/d2sc06624f

**Published:** 2023-01-09

**Authors:** Daniel H. Lysak, Flavio V. C. Kock, Salvatore Mamone, Ronald Soong, Stefan Glöggler, Andre J. Simpson

**Affiliations:** a Environmental NMR Centre, University of Toronto Scarborough 1265 Military Trail Scarborough Ontario Canada; b Department of Chemistry, Federal University of São Carlos (UFSCar) Rod. Washington Luís, Monjolinho São Carlos–SP 13565-905 Brazil; c NMR Signal Enhancement Group, Max Planck Institute for Multidisciplinary Sciences Am Fassberg 11 37077 Göttingen Germany andre.simpson@utoronto.ca stefan.gloeggler@mpinat.mpg.de

## Abstract

In line with recent paradigm shifts in toxicity testing, *in vivo* nuclear magnetic resonance (NMR) is a powerful tool for studying the biological impacts and perturbations caused by toxicants in living organisms. However, despite the excellent molecular insights that can be obtained through this technique, *in vivo* NMR applications are hampered by considerable experimental challenges such as poor line shape and spectral overlap. Here, we demonstrate the application of singlet-filtered NMR to target specific metabolites and facilitate the study of metabolite fluxes in living *Daphnia magna*, an aquatic keystone species and model organism. Informed by mathematical simulations and experiments on *ex vivo* organisms, singlet state NMR is used to monitor the flux of metabolites such as d-glucose and serine in living *D. magna*, during the environmentally relevant processes of anoxic stress and reduced food availability. Overall, singlet state NMR is shown to have significant future potential for studying metabolic processes *in vivo*.

## Abstract 2

Using a singlet state filter, nuclear magnetic resonance (NMR) can selectively monitor metabolites, while suppressing undesired signals in living *Daphnia magna*, allowing metabolite tracking even in regions of significant spectral overlap.

## Graphical abstract

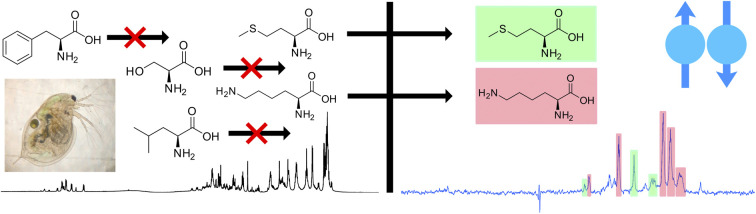

## Introduction

Whether used to determine the structure of an unknown compound, examine the carbon distribution of a soil or study non-covalent interactions in macromolecules, nuclear magnetic resonance (NMR) spectroscopy is a powerful analytical technique with wide ranging applications. Numerous advantages, including molecular insights allowing *de novo* structural determination, facile quantification, unrivalled reproducibility and inherently non-destructive analysis have made NMR a pillar in the field of metabolomics.^1–3^ Of particular note, the non-destructive nature of NMR opens the door towards the study of organisms *in vivo*.^4^ This ability to study living organisms allows for the monitoring of metabolic changes *in vivo*, for example in response to changing environmental conditions such as decreased oxygen or food, presence of pollutants or varying pH, has resulted in increasing interest in this technique for the purposes of studying toxicity.^5^

The landmark report *Toxicity testing in the 21st century: a vision and a strategy*, commissioned by the U.S. National Academy of Sciences, noted that traditional toxicity testing “relies primarily on apical endpoints (e.g. death, loss of movement) upon exposure to high doses of a test chemical”.^6^ However, these tests “provide little to no information on the toxic modes of action and sublethal toxicity – and a paradigm shift towards examining biological perturbations as opposed to apical endpoints is required”.^6^ When one considers that, in the environment, pollutants are rarely found in the concentrations used in acute toxicity tests,^5^ it becomes clear that such sub-lethal insights are invaluable for understanding toxicity in the real world, as well as developing effective environmental regulations.^4^

Nuclear magnetic resonance is uniquely poised to help address these existing knowledge gaps. Specifically, *in vivo* NMR has the ability to examine the metabolic profile of a living organism upon exposure to sublethal toxicant concentrations in real-time and even the potential to examine recovery in the same organisms, after the stressor is removed.^5^ However, despite the exceptional potential and considerable previous success of this technique, there are significant experimental challenges faced *in vivo*. Of note, the line shapes resulting from an *in vivo* spectrum are typically much broader than for true solutions, due to the differences in magnetic susceptibility caused by the different “compartments” of the organism.^5^ Thus, when combined with the inherent natural complexity of a living organism, along with the fact that lipids often dominate the ^1^H spectral envelope, it becomes difficult to isolate metabolite signals directly from ^1^H NMR.^7^

One solution, that has been applied for the study of small aquatic organisms in an environmental context, has been to culture organisms on a purely ^13^C diet and then use the increased signal to obtain heteronuclear 2D ^1^H–^13^C spectra that provide the additional spectral dispersion required to assign and monitor metabolites *in vivo*.^7^ While an elegant solution, the approach limits studies to organisms raised in the lab and is prohibitively expensive over the long term. On the other hand, highly selective NMR approaches have been introduced, that allow multiple targets inside organisms to be isolated and monitored.^8^ However, such approaches are challenging to implement and involve the generation of tailored waveforms that must be changed for every metabolite or metabolite combination. On the other hand, singlet state NMR provides the potential to isolate signals without the need for any selective excitation, offering a simple and robust approach for targeted *in vivo* monitoring.

Singlet states are effective spin 0 states that can be created between spin pairs. They received increased attention as soon as it was realized that they can persist for longer times (up to hours) compared to longitudinal magnetization states in favourable situations, depending on the molecular structure and spin network.^9–11^ Over time, they have been proposed as a tool for studying slow diffusion,^12–14^ drug binding and protein folding,^15^ self-assembling and stimuli-response phenomena,^16–18^ and storage for signal in hyperpolarization.^19,20^ More recently, singlet states have been proposed as quantum filters to increase the contrast of certain resonances from undesired background signals.^21–24^

In this work the gc-M2S2M sequence was used to bring longitudinal magnetization into the singlet state and back^24^ (see the ESI† for experimental details and simulations). The gc-M2S2M sequence can generate singlet states in spin pairs in any coupling regime by appropriate settings of the sequence parameters. It is insensitive to B_0_ inhomogeneities and pulse offsets (within the bandwidth of the pulses). The signal selectivity depends strongly on chemical shift differences and spin J-couplings, and it was observed that the sequence is very effective in suppressing signals that do not pass through the singlet state (as selected by the sequence parameters).

To illustrate the potential of singlet filtered NMR for improving contrast *in vivo*, we demonstrate here, to our knowledge, the first reported use of singlet filtered nuclear magnetic resonance to study metabolic changes *in vivo*. The gc-M2S2M sequence^24^ allows for selective identification of metabolites *in vivo*, and thus for monitoring of individual or small groups of metabolites during stress responses. *Daphnia magna* (water fleas) are studied, which are among the most common species for aquatic toxicity testing and are highly responsive to environmental stresses.^25,26^ Further, *D. magna* have recently been shown to be important in the transport of pollutants such as polystyrene nanoparticles through upper trophic levels, including fish that are consumed by humans.^27^*Daphnia* also provide an important link between aquatic producers such as algae and aquatic consumers such as fish^27,28^ and, as such, are considered an ecological keystone species.^7^ Studies have shown that *Daphnia* are effective model organisms for human health and disease,^29^ and have recently been listed as a National Institute of Health model organism,^26^ making their study of particular interest.

## Results and discussion

**Fig. 1:**
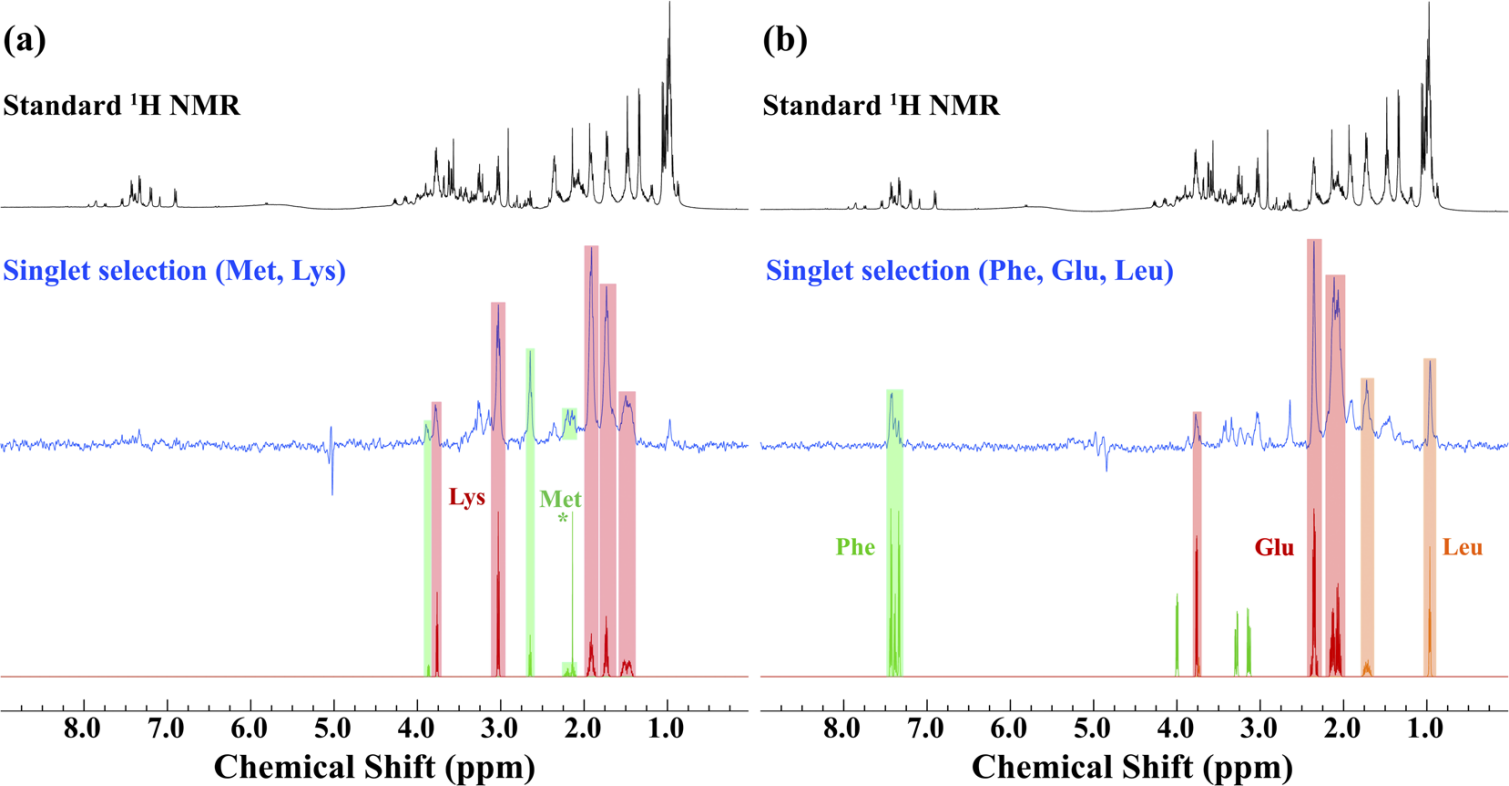
An example of singlet filtered NMR spectroscopy (at 500 MHz) on *ex vivo Daphnia magna* used for co-selection of metabolites *in vivo*. In both cases, the top spectrum shows a standard ^1^H NMR spectrum with water suppression. As the organisms have died, high concentrations of metabolites leach into the surrounding water and lipids tend to float above the coil giving excellent resolution – an ideal system to demonstrate the singlet-state concept in a biological relevant sample. The blue spectra are singlet-state targeted for (a) methionine and lysine and (b) phenylalanine, glutamic acid and leucine. The bottom panels show the corresponding compound matches from the Bruker Bio-reference database. The (*) signal represents the CH_3_ peak in methionine.

Prior to the application of the gc-M2S2M sequence to living *D. magna,* mathematical simulations (see ESI Section 2.1†) were used to help identify starting experimental conditions for the selection of individual metabolites, and the conditions were tested and optimized on *ex vivo* organisms (Fig. 1). The singlet filtering part of the sequence is defined by the following 4 parameters: the number of echoes in the single-quantum and zero-quantum block *n*_1_ and *n*_2_, the echo delay *τ* and the zero-quantum delay *Δ*. For the selected proton pairs, the efficiency *f* was determined by spanning the corresponding 4-dimensional parameter space, see Section 2.2 of the ESI† for details. In practice, signal losses may arise when the sequence duration becomes long compared to the relaxation mechanisms, that are more effective *in vivo*. Therefore, the parameters were chosen by trying to balance the theoretical transfer efficiency and the total sequence time *T* = 4*n*_1_*τ* + 2(*Δ* + *n*_2_*τ*).

We identified sets of metabolites that can be selected concurrently. This can be a considerable advantage simply for the fact that it can result in significant time savings, allowing for improved throughput, and greater information content from a single experiment. If, for example, one wishes to track a process that is known to involve changes of two metabolites, it would increase confidence if both trends can be seen in the same experiment. This concept is demonstrated first on *ex vivo D. magna* (see Fig. 1) before moving on to living *Daphnia*. In this case, the sequence was modified to contain a total correlation spectroscopy (TOCSY) mixing block and a zero-spoil block. The TOCSY block allows magnetization transfer through the selected ^1^H–^1^H spin system^30^ which aids in identification of the selected metabolite/metabolites, while the zero quantum spoil suppresses zero quantum components, improving phase and lineshape.^31^ However, application of the TOCSY block comes at the cost of sensitivity and therefore it was used only for metabolite identification, but not for monitoring. Though the loss in sensitivity is dependant on the individual spin system and mixing times chosen, a sensitivity decrease on the order of 40–60% occurs using a 120 ms mixing time for d-glucose (See Fig. S4† for more details). The full pulse sequence code is provided in the ESI,† Section 3.

Starting with lysine, the singlet state filter with TOCSY pulls out all the signals. Indeed, all the protons belonging to the spin system which are coupled to the selected singlet state sub-unit become observable. Methionine, on the other hand, contains an isolated CH_3_ group (see * in Fig. 1). This group is not spin coupled to the remaining spins that are selected by the singlet state TOCSY filter and thus is not detected. In the case of phenylalanine, the singlet state target is in the aromatic ring which is not strongly coupled to the aliphatic side chain and thus not detected. Conversely, both leucine and glutamic acid contain fully coupled spin systems and all spins in these molecules are detected. Here, we note how singlet filtration simplifies the spectrum and allows a trustworthy metabolite identification. The standard ^1^H NMR spectrum shows a wide range of overlapping peaks, while the selective spectrum is heavily biased towards the desired compounds, and signals from unwanted compounds are strongly (although not completely) suppressed.

**Fig. 2:**
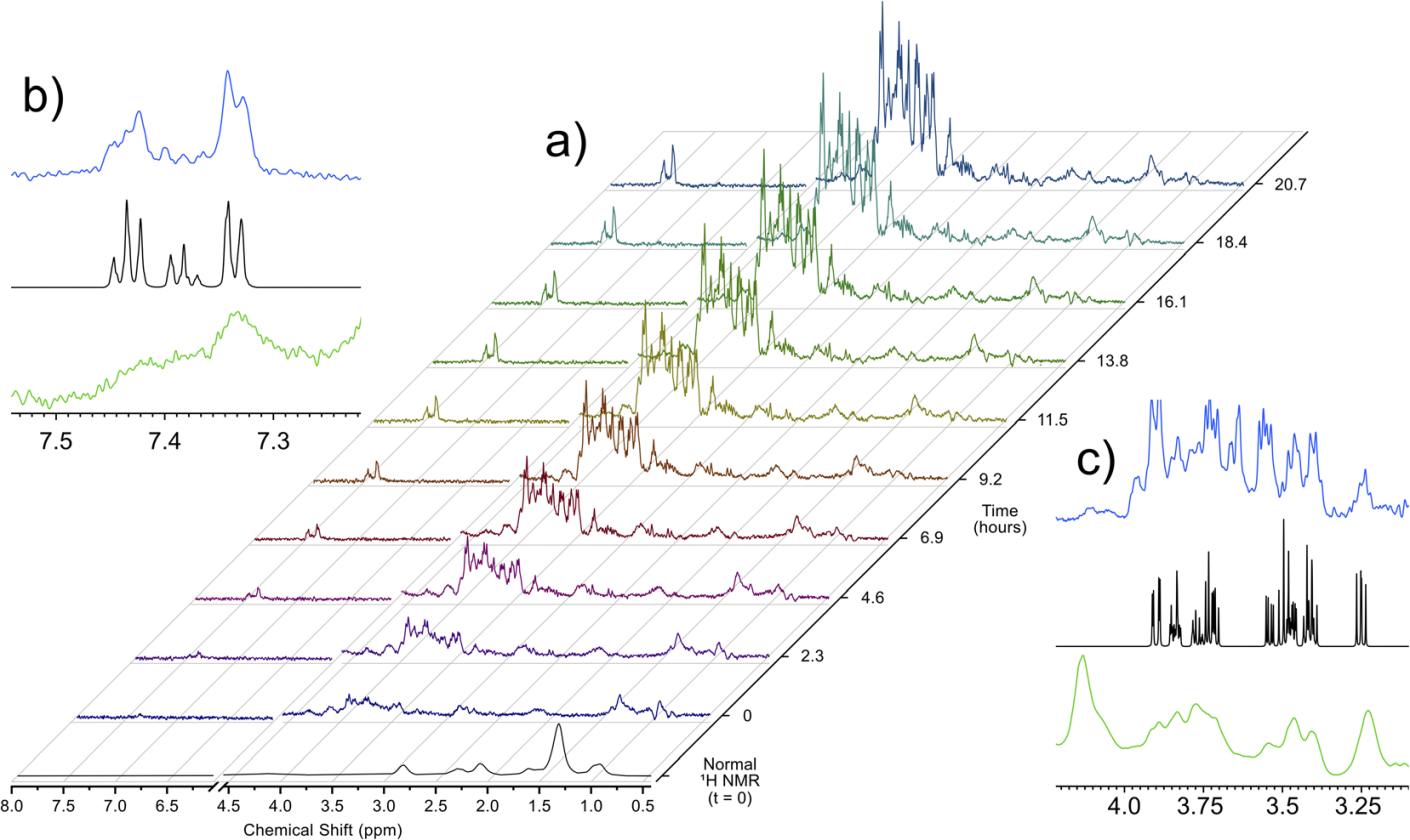
(a) Increase in glucose and phenylalanine concentrations in *in vivo D. magna* under anoxic stress as monitored by singlet filtered NMR at 500 MHz. A standard ^1^H NMR spectrum at *t* = 0 is provided for comparison. Insets (b) and (c) show the singlet filtered spectra (blue) and standard ^1^H spectrum (green) at 20.7 hours for phenylalanine and glucose, along with the corresponding Bruker Bio-reference database spectra (black).

Utilizing the parameters that were optimized through simulations and validated on *ex vivo* samples, the singlet state sequence was applied to trace the process of anoxic stress *in vivo*. For a typical *in vivo* experiment, the organisms are sustained in a 5 mm flow system, which provides food, oxygenated water, and removes waste products. This system, described in a previous work,^32^ allows for *D. magna* to be sustained under low stress inside the spectrometer indefinitely. Turning off the flow induces anoxic stress in the organisms and Fig. 2 shows a time lapse of a singlet filtered experiment throughout this process. Fig. S3† shows expanded spectral regions demonstrating that without the singlet-filter the metabolites of interest cannot be identified. Here, the target compounds were: glucose, which has a central role in energy metabolism, and phenylalanine, an amino acid that is important as a protein building block as well as a precursor to numerous signalling molecules such as dopamine.^33^ As can be seen in the insets in Fig. 2, the singlet filtered spectrum shows an excellent match to the target metabolite spectrum, despite the considerable matrix effects that can influence *in vivo* data.^5^ Neither compound can be discerned in the standard ^1^H NMR *in vivo* data (first spectrum in Fig. 2a). The increase in both phenylalanine and glucose concentrations as time progresses is a result of the biological response to increasingly severe anoxia. As the oxygen concentration in the water decreases, the potential for aerobic respiration is reduced, and the organism breaks down energy storage molecules (*i.e.*, glycogen) into glucose in order to perform anaerobic glycolysis.^34^ The increase in phenylalanine can be attributed to broad-scale protein breakdown, which occurs in order to provide free amino acids, that are in turn used for energy production.^35,36^

**Fig. 3:**
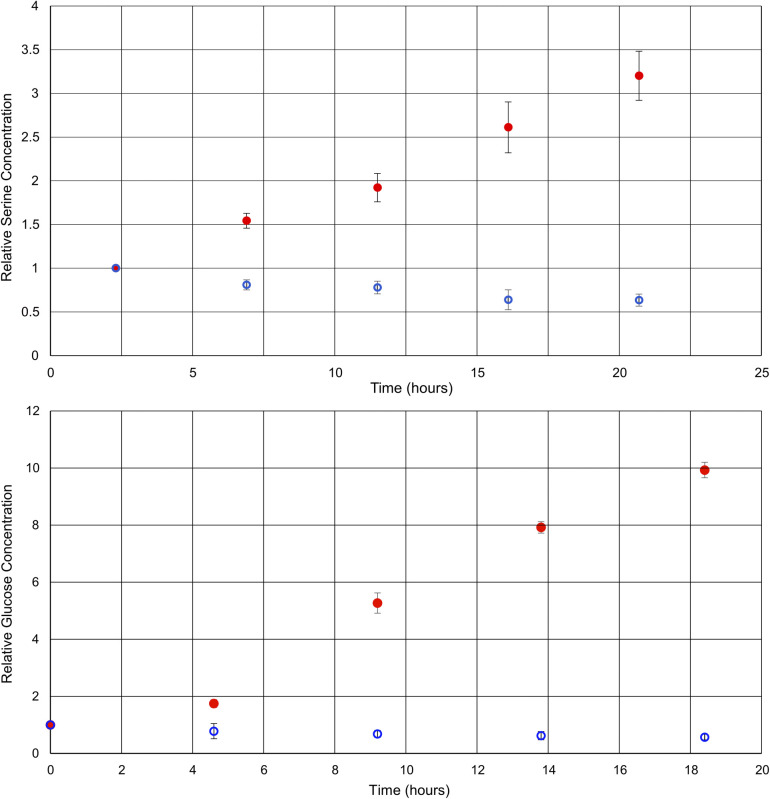
Graphs of the relative concentrations of serine and glucose through the process of anoxic stress (red) and halved food availability (blue). Each experiment was repeated in triplicate.

By comparing the intensities of the selected peaks across spectra acquired under the same conditions, it is possible to quantify metabolite changes on a relative basis. Although relative quantification can be performed quite easily, due to the variations in singlet transition efficiency for different spin systems, absolute quantification is more challenging. For absolute quantification a standard addition of the target compound would be required. This approach has been previously described in the literature for other selective NMR experiments.^8^Fig. 3 shows plots of the relative concentrations of glucose and serine *in vivo*, throughout two different environmental conditions: anoxic stress (red data points) and decreased availability of nutrients (blue data points).

Here, experiments focusing on serine and glucose were interleaved such that both could be monitored and repeated in triplicate. As such, the time axes for the metabolites are staggered and the temporal resolution reduced in comparison to Fig. 2. Phenylalanine was not quantified, as the initial concentration was below the limits of quantification at time zero in some of the *D. magna* samples. In the case of the anoxic stress experiment, the first data point is gathered with the flow system on, and it is turned off for subsequent data points. As expected, anoxia has a large impact on the metabolite concentrations.^7^ Glucose increases due to anaerobic glycolysis^34^ and serine increases due to protein breakdown to provide free amino acids for energy production^35,36^

In contrast, the reduction of food over a relatively short period (24 h) has been documented to have a much smaller impact,^37^ making this a test of more nuanced metabolite tracking. In this case, the flow system was kept on for the duration of the experiment, but the tank water was diluted in half with dechlorinated tap water. The decreased food availability caused by the halving of the algae concentration can be expected to cause a decrease in short term energy stores such as glucose, as metabolism continues, but the opportunity for replenishment is decreased. Similarly, the decrease in serine concentration may be attributed to its role as a precursor to pyruvate,^38^ which feeds into the Krebs cycle.^36^

## Conclusions

In conclusion, singlet-filtered NMR has been shown, for the first time, to be able to track a metabolic response to stressors *in vivo*. Specifically, singlet filtered NMR allowed the monitoring of selected metabolites *in vivo* in both an acute (anoxic stress) and chronic (reduced food) scenario and holds promise for future studies to better understand various biological processes including growth, toxic responses and recovery inside living organisms. The selection and detection of multiple metabolites has been demonstrated as well. This technique yields significantly simplified NMR spectra with reduced overlap and has considerable potential for non-destructive *in vivo* metabolomics. In the future, this technique could be used to examine the effects of and recovery from exposure to environmentally relevant toxicants such as polyfluorinated compounds, pesticides or microplastics. Further, the analytes studied here are of considerable importance to human health: glucose imbalance is, of course, integral in diabetes and can be a biomarker for various types of cancer,^39^ phenylketonuria is caused by the inability to breakdown phenylalanine,^40^ and serine is a metabolic precursor to glutathione, which is responsible for defense against reactive oxygen species.^41^ As such, using these compounds, there are numerous additional metabolic pathways that are impactful for human health that could be studied, demonstrating considerable potential for this approach.

## Data availability

The pulse program and associated code are provided in the ESI.† Example data sets are available on request.

## Author contributions

AJS and SG supervised the project and were responsible for funding acquisition. DHL and FVCK prepared, optimized and performed the NMR experiments. SM performed the singlet state simulations. DHL, SM and FVCK contributed to formal analysis, data curation and validation. DHL contributed to data visualization and wrote the original draft. All authors contributed to conceptualization of the project and editing/review of the manuscript.

## Conflicts of interest

There are no conflicts to declare.

## Supplementary Material

SC-014-D2SC06624F-s001
