# Supplementary material for: In vivo singlet state filtered nuclear magnetic resonance: towards monitoring toxic responses inside living organisms
Source: Chem Sci. 2023 Jan 9;14(6):1413–8. doi: 10.1039/d2sc06624f (PMC9906653; doi:10.1039/d2sc06624f)
Supplement: SC-014-D2SC06624F-s001 [file SC-014-D2SC06624F-s001.pdf]

Electronic Supplementary Material (ESI) for Chemical Science.
This journal is © The Royal Society of Chemistry 2023

Electronic Supplementary Material (ESI) for Chemical Science.
This journal is © The Royal Society of Chemistry 2023

Electronic Supplementary Information for:

In vivo Singlet State Filtered Nuclear Magnetic Resonance: Towards Monitoring Toxic Responses inside Living Organisms

Daniel H. Lysak,[‡a] Flavio V. C. Kock,[‡b] Salvatore Mamone,[‡c] Ronald Soong,[a] Stefan Glöggler*[c] and Andre J. Simpson[*c]

[a]Environmental NMR Centre, University of Toronto Scarborough, 1265 Military Trail, Scarborough, Ontario, Canada
E-mail: andre.simpson@utoronto.ca
[b]Department of Chemistry, Federal University of São Carlos (UFSCar), Rod. Washington Luís, Monjolinho, São Carlos - SP, 13565-905, Brazil
[c]NMR Signal Enhancement Group, Max Planck Institute for Multidisciplinary Sciences, Am Fassberg, 11 37077, Göttingen, Germany
E-mail: stefan.gloeggler@mpinat.mpg.de
‡These authors contributed equally.
[*]Corresponding authors

Table of Contents

1. Experimental Procedures 2
2. Additional Discussion 3
   2.1 Discussion of the Singlet State Filtered Pulse Sequence 3
   2.2 Discussion of Singlet State Simulations 3
   2.3 Comparison of Singlet Filtered Spectra to a Standard ^{1}H Spectrum 4
3. Appendix A: Singlet State Pulse Sequence for Bruker Spectrometers (TS3 and TS4) 5
4. References 10

1. Experimental Procedures

Unless otherwise indicated, all chemicals were purchased from Sigma Aldrich (St. Louis, Missouri, U.S).

Organism Culturing

Daphnia magna, originally purchased from Ward's Science Canada, (St Catharines, ON, Canada) have been cultured in house for the last 8 years. The culture conditions were as follows: a 16:8 hour light cycle, ambient temperature in dechlorinated municipal tap water (aged at least one week, and aerated constantly prior to use). The organisms were fed a diet of fresh algae (*Chlamydomonas reinhardtii*) which was cultured in house, three times a week, and a 50% water change was performed at the time of feeding. The algae were cultured in Bristol medium and concentrated via centrifugation before use. *Daphnia magna* are small freshwater fleas. They are invertebrates and not considered animals with respect to ethics protocols that apply to higher order vertebrates. In Canada no ethics approvals are required for the study of these invertebrates, this has been confirmed in writing by the university ethics committee.

Ex vivo *D. magna* Sample Preparation

To prepare the ex vivo sample, 30 adult *D. magna* were removed from the culture medium, frozen with liquid nitrogen, and lyophilized overnight. Prior to NMR analysis, the lyophilized organisms were placed into a 5 mm NMR tube, reconstituted in 90% water and 10% D_2O, and crushed by manual agitation. 0.5% W/W sodium azide was added to prevent bacterial degradation, and NMR experiments were carried out at 5°C, to further decrease degradation.

In Vivo NMR Experiments

The organisms were sustained by a 5 mm flow system as previously described[1]. 30 adult *D. magna* were placed in a 5 mm NMR tube, along with a capillary filled with D_2O to act as a lock. The experiments were carried out at 5°C in order to improve survivability. For the reduced nutrient experiments, the tank water was diluted in half with fresh, oxygenated, dechlorinated water, and the flow system was kept on for the entire duration. For the anoxic stress experiments, the flow system was on for the first data point, and then turned off afterwards.

NMR Spectroscopy - General Methods

Ex vivo experiments were performed on a Bruker Avance III 500 MHz (^{1}H) NMR spectrometer using a 5 mm Bruker ^{1}H-^{13}C-X TBI room temperature probe fitted with an actively shielded z-gradient, and a deuterium lock channel. In vivo experiments were performed on a Bruker Avance III HD 500 MHz (^{1}H) NMR spectrometer using a 5 mm ^{1}H-^{13}C-^{15}N TCI CryoProbe Prodigy fitted with an actively shielded z-gradient, and a deuterium lock channel.

Prior to experimental acquisition, each channel used was tuned and matched, and the 90° pulse was calibrated (typical values ranged from 9-10 µs) on a per sample basis. All spectra were phased, baseline corrected, and processed using an exponential function corresponding to a line broadening of 1 Hz and zero-filling factor of 2. All data were acquired using Topspin version 3.5pl5. The spectra were referenced to known compounds in the Bruker Bioreference databases (versions 2.0.1 to 2.0.5).

NMR Spectroscopy - Standard 1D ^{1}H NMR Experimental Parameters

The 1D ^{1}H NMR spectra which were compared to the singlet filtered spectra (see Fig. 2) were acquired using presaturation and water suppression by gradient-tailored excitation with W5 pulse trains (W5 WATERGATE)[2,3] with a binomial delay of 125 µs. Data were acquired using a spectral width of 16.4 ppm, 32 scans, 8 dummy scans, 8192 time domain points, and a recycle delay of 2 s.

NMR Spectroscopy - Singlet State (SS) Filtered Experimental Parameters

The 1D SS ^{1}H NMR spectra were acquired using the gc-M2S2M sequence as described in section 2.1 of the Supplementary Information. Data were acquired with a spectral width of 16.4 ppm, 3072 scans, 8 dummy scans, 8192 time domain points, and a recycle delay of 2 s. The multi-integration feature of MestReNova 12.0 was used to calculate the integrals used to generate Fig. 3. The 4 singlet state parameters used for each experiment are outlined in Table S1. For ex vivo experiments a TOCSY mixing time of 120 ms was used, but this was turned off to enhance signal for in vivo experiments.

Table S1. A list of the experimental singlet state parameters used for each singlet filtered experiment.

Parameter	Ex vivo (methionine, lysine)	Ex vivo (phenylalanine, glutamic acid, leucine)	In vivo (glucose, phenylalanine)	In vivo (serine)
n_1	2	1	4	1
n_2	1	3	4	1
Δ (ms)	26	5	6.5	11.5
τ (ms)	31	10	4.75	19

2. Additional Discussion

2.1 Discussion of the Singlet State Filtered Pulse Sequence

In this work, the gc-M2S2M sequence is used to bring longitudinal magnetization into the singlet state and back[4]. For metabolite identification, the sequence is followed by a TOCSY block to transfer the filtered magnetization through the coupled spin network, before signal acquisition, see Fig. S1. The singlet filtering part of the sequence is defined by the following 4 parameters: the number of echoes in the single-quantum and zero-quantum block n_1 and n_2, the echo delay τ and the zero-quantum delay Δ.

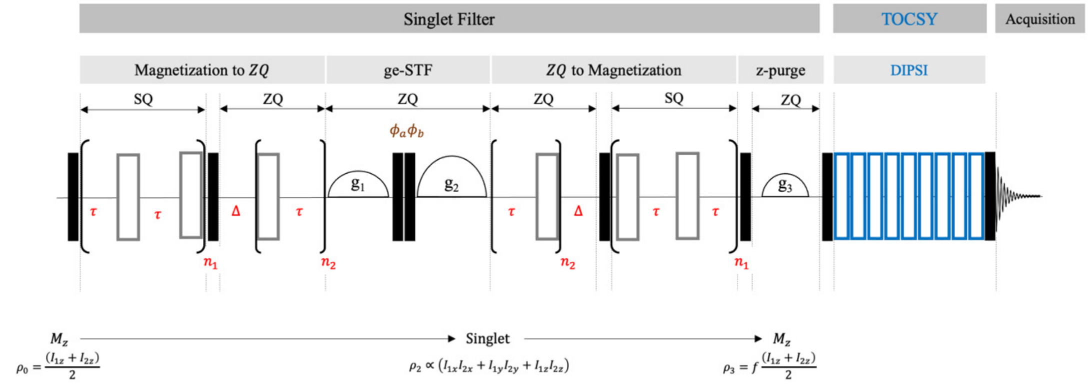

Fig. S1. The pulse sequence used to filter the signal from metabolites is shown here. It consists of a singlet filter block that excites only specific ^{1}H pairs in the selected metabolite (with efficiency f compared to a single hard pulse) by tuning the experimental parameters τ, n_1, Δ, n_2. The TOCSY block uses DIPSI pulses to spread the magnetization over the network of coupled protons in the molecule. Optional W5 and presaturation blocks are standard and are not shown for clarity. A copy of the pulse program can be found in section 3 of the Supplementary Information.

2.2 Discussion of Singlet State Simulations

For the set of selected metabolites, the ^{1}H chemical shifts and couplings were retrieved from literature (glucose, glutamic acid, phenylalanine, serine)[5] or extracted from fitting the spectrum in the HMDB (L-methionine, L-lysine and L-leucine). In setting the spin system, all the couplings to exchangeable protons were neglected. All the simulations were performed at 500 MHz. The power level for pulses was set to a nutation frequency of 25 kHz, corresponding to a 90° hard pulse of 10 µs. Gradients were simulated as zero-quantum projectors. The gradient-enhanced spherical tensor T_{00} filter (ge-STF) used here is based on the concept of spherical tensor analysis (STA).[6] It is implemented as a pair of 90° hard pulses flanked by gradients, with the phases for the first STA pulse set always to zero and for the second 90° STA pulse cycling in 4 steps {37.38, 79.19, 217.38, 259.19}[7] degrees. The delays before and after the STA include the gradients pulses. These were set to 300 µs. The effects of relaxation were disregarded in the simulations.

For the selected protons of interest, the efficiency f for the operator transformation $\sum_i I_{z,i} \to f \sum_i I_{z,i}$ (with i spanning the spin of interest for singlet transfer) was determined by performing a grid search on the 4-dimensional parameter space $\{n_1, n_2, \tau, \Delta\}$. The grid was defined according to the following scheme:

$$n_1 \in Range[1, n_{1,max}, 1]$$
$$n_2 \in Range[0, n_{2,max}, 1]$$
$$\tau \in Range[1, \tau_{max}, \tau_{inc}]$$
$$\Delta \in Range[0, \Delta_{max}, \Delta_{inc}]$$

The total number of simulations was $N_{sim} = n_{1,max} \times (n_{2,max}+1) \times \left(\frac{\tau_{max}-\tau_{min}}{\tau_{inc}}\right) \times \left(\frac{\Delta_{max}-\Delta_{min}}{\Delta_{inc}}\right)$. Specifically for glucose $n_{1,max}$=$n_{2,max} = 5, \tau_{max} = \Delta_{max} = 20$ ms, $\tau_{inc} = \Delta_{inc} = 0.25$ ms, for a total of ~200 x 10^3 simulations; for glutamic acid, phenylalanine and serine $n_{1,max}$=$n_{2,max} = 5, \tau_{max} = \Delta_{max} = 50$ ms, $\tau_{inc} = \Delta_{inc} = 0.5$ ms for a total of ~300 x 10^3 simulations; for methionine, lysine and leucine, $n_{1,max} = n_{2,max} = 3$, $\tau_{max} = \Delta_{max} = 50$ ms, $\tau_{inc} = \Delta_{inc} = 1$ ms for a total of ~30 x 10^3 simulations. For each metabolite, a two-dimensional result matrix was built with rows and columns given by n_1 and n_2 and for entries the values τ and Δ and the corresponding efficiencies f and total timings $T = 4n_1\tau + 2(\Delta + n_2\tau)$. The parameters used for the actual experiments are shown in Table S2. They were chosen by trying to retain high efficiency, short total sequence time T and filter selected metabolites simultaneously (on one side glutamic acid, phenylalanine, leucine and on the other side methionine and lysine). A typical efficiency contour plot for fixed n_1 and n_2 for the 6,6' proton in the alpha and beta anomer of glucose is shown in Fig. S2.

Table S2. A summary of the parameters used for singlet filtration of the signal of the indicated spin pairs and the corresponding theoretical efficiencies.

Metabolite	Singlet protons	n_1	n_2	τ	Δ	f
Glucose (α anomer)	^{6}CH-$^{6'}$CH	2	2	11	18.5	30%
Glucose (β anomer)	^{6}CH-$^{6'}$CH	4	4	4.75	6.5	36%
Glutamic Acid	$^{\beta}CH_2$	1	3	10	5	13%
Phenylalanine	^{2}CH-^{5}CH<br>^{3}CH-^{6}CH (phenyl ring)	1	3	10	5	3.3%
Serine	$^{\beta}CH_2$	1	1	19	11.5	35%
Methionine	$^{\gamma}CH_2$	2	1	31	26	11.5%
Lysine	$^{\beta}CH_2$	2	1	31	26	1.2%
Leucine	$^{\beta}CH_2$	1	3	10	5	2.8%

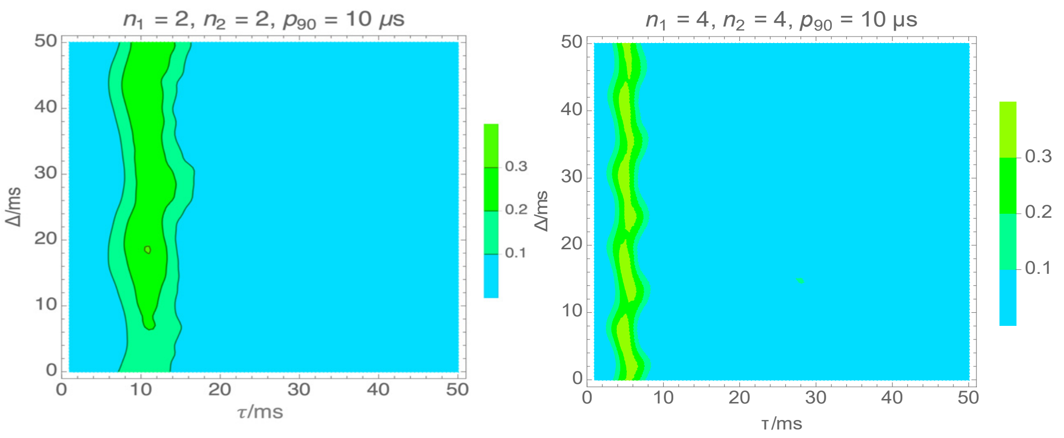

Fig. S2. Contour plot of the efficiency of magnetization to singlet to magnetization conversion of the gc-M2S2M sequence for the ^{6}CH-$^{6'}$CH in the alpha anomer (panel a) and beta anomer (panel b) of glucose as a function of τ and Δ for fixed n_1 and n_2. In both cases, the dependence on Δ is weak across the explored range.

2.3 Comparison of Singlet Filtered Spectra to a Standard ^{1}H Spectrum

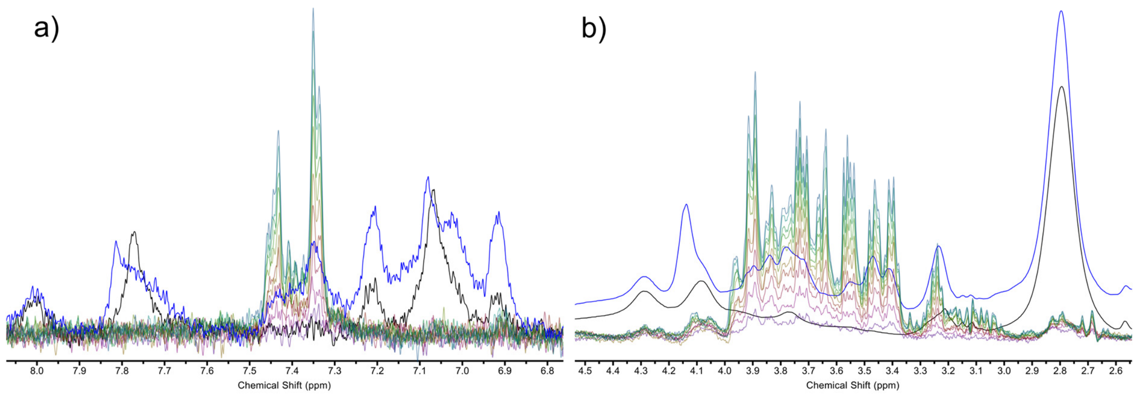

Fig. S3. A comparison of the time series ranging from 0 to 20.7 hours (coloured spectra) for in vivo *D. magna* undergoing anoxic stress (see Fig. 2 for more details), to a standard ^{1}H NMR spectrum at t = 0 (black) and t = 20.7 (dark blue). a) shows the aromatic region with singlet selection for phenylalanine, while b) shows the carbohydrate region with singlet selection for glucose. Neither of these metabolites are distinguishable in the standard ^{1}H spectrum.

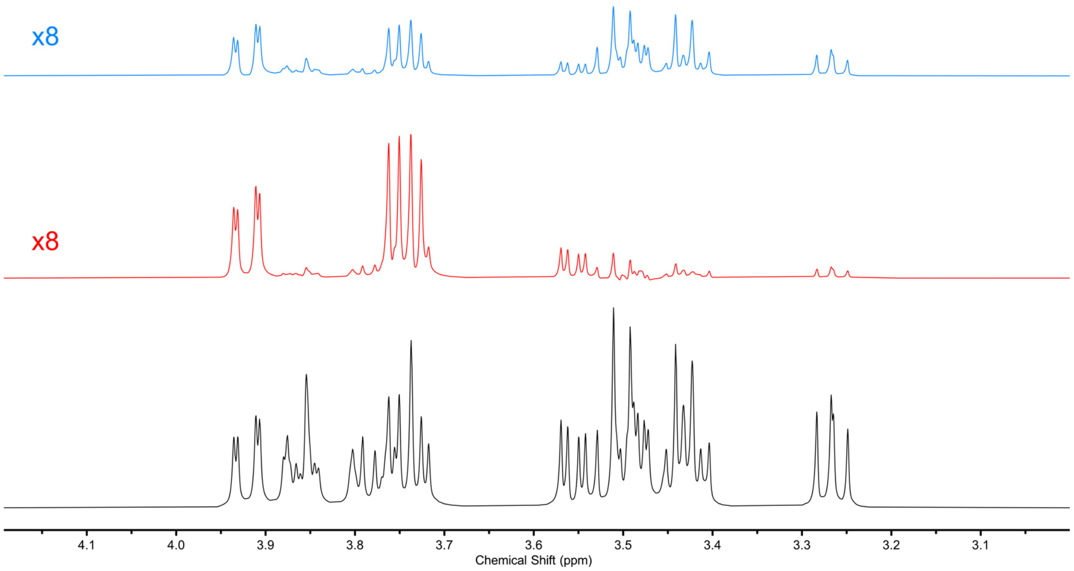

Fig. S4. A comparison of a reference ^{1}H spectrum (black) to a singlet filtered spectrum with (blue) and without (red) a TOCSY mixing time of 120 ms for 100 mM D-glucose. The singlet state has been optimized to select the β anomer of D-glucose. Using the parameters for β-glucose from Table S2, ~17-18% of signal is retained after the application of the singlet state sequence, and this value drops to ~8-9% with TOCSY (120ms). The red and blue spectra have been scaled up to better illustrate the changes with and without TOCSY.

3. Appendix A: Singlet State Pulse Sequence for Bruker Spectrometers (TS3 and 4)

```
#include <Avance.incl>
#include <Grad.incl>
#include <Delay.incl>

;simplified T00 filter

;SQ echoes are in the form 180-delay-180-delay
;ZQ echoes are in the form  180-delay
;composite 180 pulse is 180_0->(60_180)(300_0)(60_180)
;added phase arithmetic for composite 180
;

;z-purge block at the end of the sequence

;d1: inter-scan delay
;p1: 90 degree pulse duration at power level PLW1

"FACTOR1=(d9/(p6*115.112))/2"
"l1=FACTOR1*2"

"p27=p1"
"plw18=plw1"

"acqt0=-p1*2/3.1416"

"cnst15=o1"

;p1: 90 degree pulse duration at power level pl1
;pl9: presat power level pl9
"p2=2*p1"

;;sequence parameters           begin

; d21: half-echo duration (optimize experimentally )
; d20: ZQ free pre-echo train evolution  (optimize experimentally )
; l21: number of lopps for SQ blocks (optimize experimentally )
; l22: number of loops for ZQ blocks (optimize experimentally )

;;S2M parameters end

;; composite 180 pulses 180_0->(60_180)(300_0)(60_180)
"p21=(2.0/3.0)*p1"
"p22=(10.0/3.0)*p1"


;d14: gradient recovery in T00
```

```
;d14 is set to 100u here
;"d14=100u"



;singlet evolution
;d15:  singlet sustaining period
;CPDPRG15:  singlet sustaining CPD
;plw15  power for pulses during the sustaining period
;cnst15: singlet sustaining  RF offset (ppm)
;(fixed to CALCULATED o1p or adjust commenting below)
"cnst15=(1e6)*(sfo1-bf1)/bf1"
"cnst25=cnst15-(1e6)*(sfo1-bf1)/bf1"

;z-filter
;p29:    duration of the z-purge gradient
;gpz29: gradient strength z-purge
;d29;    z-purge delay

1 ze



2 30m
  d12 pl9:f2
  d1 cw:f2 ph29
  4u do:f2
  4u pl1:f1

;;;;;;;;;;;;;;;;;;M2S;;;;;;;;;;;;;;;;;
;;;;;;;1st M2S block;;;;;


;;;;;;;;SQ in phase from equilibrium I1z+I2z;;;;;;;;;
0.5u fq=0.0:f1
(p1 pl1 ph10):f1

;;;;;;;;;;;;;;;;;;M2S;;;;;;;;;;;;;;;;;

;;;;;; M2S-SQ block;;;;;

21
d21
(p21 pl1 ph21):f1
(p22 pl1 ph22):f1
(p21 pl1 ph21):f1
d21
(p21 pl1 ph21+180):f1
(p22 pl1 ph22+180):f1
(p21 pl1 ph21+180):f1
lo to 21 times l21

(p1 pl1 ph11):f1                                                        ; convert SQ into ZQ

;;;;;;; M2S-ZQ block ;;;;;
d20
22
(p21 pl1 ph25):f1
(p22 pl1 ph26):f1
(p21 pl1 ph25):f1
d21
lo to 22 times l22

;;;;; T00 filter begin ;;;;;
; gradient before STA
10u UNBLKGRAD
p13:gp13
d14

; STA pulses begins
(p1 pl1 ph16):f1
(p1 pl1 ph17):f1
; STA pulses end

;gradient after STA pulses
p13:gp14
d14 BLKGRAD


;;;;; T00 filter ends
```

```
;;;;;Evolution block begins
#ifdef _ss
0.5u fq=cnst25:f1
1u pl15:f1
(1u cpd1 ph15):f1
d15
1u do:f1
1u pl1:f1
0.5u fq=0.0:f1
#endif
;;;; evolution block ends



;;;;;;;;;;;;;;;;;S2M;;;;;;;;;;;;;;;;

;;;;;; S2M-SQ;;;;;
23
d21
(p21 pl1 ph27):f1
(p22 pl1 ph28):f1
(p21 pl1 ph27):f1
lo to 23 times l22
d20



(p1 pl1 ph12):f1                                                    ;covert ZQ into SQ

;;;;;S2M-ZQ;;;;;

24
(p21 pl1 ph23):f1
(p22 pl1 ph24):f1
(p21 pl1 ph23):f1
d21
(p21 pl1 ph23+180):f1
(p22 pl1 ph24+180):f1
(p21 pl1 ph23+180):f1
d21
lo to 24 times l21

;;;;;; z-purge ;;;;;;;;;;;;;
(p1 pl1 ph13):f1


#   ifdef _ZQS ; zero quantum spoil
   10u gron0
 (p32:sp29 ph14):f1
  20u groff
  d16
#   else

#   endif /*_ZQS*/

#   ifdef _TOCSY ; optional TOCSY

  4u pl10:f1
            ;begin DIPSI2
4 p6*3.556 ph7
  p6*4.556 ph9
  p6*3.222 ph7
  p6*3.167 ph9
  p6*0.333 ph7
  p6*2.722 ph9
  p6*4.167 ph7
  p6*2.944 ph9
  p6*4.111 ph7

  p6*3.556 ph9
  p6*4.556 ph7
  p6*3.222 ph9
  p6*3.167 ph7
  p6*0.333 ph9
  p6*2.722 ph7
  p6*4.167 ph9
  p6*2.944 ph7
  p6*4.111 ph9

  p6*3.556 ph9
  p6*4.556 ph7
  p6*3.222 ph9
```

```
 p6*3.167 ph7
 p6*0.333 ph9
 p6*2.722 ph7
 p6*4.167 ph9
 p6*2.944 ph7
 p6*4.111 ph9

 p6*3.556 ph7
 p6*4.556 ph9
 p6*3.222 ph7
 p6*3.167 ph9
 p6*0.333 ph7
 p6*2.722 ph9
 p6*4.167 ph7
 p6*2.944 ph9
 p6*4.111 ph7
 lo to 4 times l1
                                                                   ;end DIPSI2
#   else
#   endif /*_TOCSY*/

#   ifdef _ZQS ; zero quantum spoil
 p16:gp3
 d16 pl10:f1
       10u gron0*1.333
 (p32*0.75:sp29 ph14):f1
 20u groff

#   else
  p16:gp3
  d16
#   endif /*_ZQS*/


 4u pl1:f1
 (p1 pl1 ph14):f1


#   ifdef _W5 ; turn on W5
 50u pl18:f2 ; begin W5
 p16:gp11
 d16
 (p27*0.087 ph2):f2
 d19*2
 (p27*0.206 ph2):f2
 d19*2
 (p27*0.413 ph2):f2
 d19*2
 (p27*0.778 ph2):f2
 d19*2
 (p27*1.491 ph2):f2
 d19*2
 (p27*1.491 ph3):f2
 d19*2
 (p27*0.778 ph3):f2
 d19*2
 (p27*0.413 ph3):f2
 d19*2
 (p27*0.206 ph3):f2
 d19*2
 (p27*0.087 ph3):f2
 50u
 p16:gp11
 d16



  4u



 p16:gp12
 d16
 (p27*0.087 ph4):f2
 d19*2
 (p27*0.206 ph4):f2
 d19*2
 (p27*0.413 ph4):f2
 d19*2
 (p27*0.778 ph4):f2
 d19*2
```

```
 (p27*1.491 ph4):f2
 d19*2
 (p27*1.491 ph5):f2
 d19*2
 (p27*0.778 ph5):f2
 d19*2
 (p27*0.413 ph5):f2
 d19*2
 (p27*0.206 ph5):f2
 d19*2
 (p27*0.087 ph5):f2
 p16:gp12
 d16

#   else

#   endif /*_W5*/

 go=2 ph31
 30m mc #0 to 2 F0(zd)
 20u BLKGRAD
exit
;;;;;Acquisition


;exit

;;;;;;;;;;;;;;;;;;;;;;;;;;;;;;;;;RF pulse phases;;;;;;;;;;;;;

ph2=0
ph3=2
ph4=0
ph5=2
ph6=1

ph8= 1

ph7=1 1 ;2 2
ph9=3 3 ;0 0

;;;; 1st 90 degree excitation pulse
ph10= {1 1 1 1}^2
;;;;; M2S 90-degree pulse
ph11= {{0 0 0 0}*2}^2
;;;;; S2M 90-degree pulse
ph12= {{0 0 0 0}*4}^2
;;;; z-filter and 90 degree acquire pulse



ph13= {{1 1 1 1}*8}^2
ph14= {{1 1 1 1}*16}^2

ph29=0

ph31= {{{{{2 2 2 2}^2}^2}^2}^2}^2


;;;phases for composite SQ1
ph21 = (360) {180 180 180 180}^2
ph22 = (360) {0 0 0 0}^2

;;;phases for composite SQ2
ph23 = (360) {{0 0 0 0}*4}^2
ph24 = (360) {{180 180 180 180}*4}^2

;;;phases for composite ZQ1
ph25 = (360) {{180 180 180 180}*2}^2
ph26 = (360) {{0 0 0 0}*2}^2
;;;phases for composite ZQ2
ph27 = (360) {{0 0 0 0}*2}^2
ph28 = (360) {{180 180 180 180}*2}^2

;;phases for evenpre

ph16 = (3600) {0 0 0 0}
;ph17=  (3600) {374 2174 1008 2808}
ph17= (3600) {374 792 2174 2592}


;phase for singlet sustaining
ph15 = {0}*128 {2}*128
```

;pl10: f1 channel - power level for TOCSY-spinlock
;pl15 power for pulses during the sustaining period
;pl18: f1 channel - power level for W5-pulse (watergate) (same as plw1)
;sp29: f1 channel - adiabatic pulse
;d9 : TOCSY mixing time
;d14 : p13 + d14 = short as possible (300us in calculations)
;d16: delay for homospoil/gradient recovery
;d19: delay for binomial water suppression
;   d19 = (1/(2*d)), d = distance of next null (in Hz) (125 us at 500 MHz)
;p6 : f1 channel - 90 degree low power pulse
;p13: homospoil/gradient pulse   [200-300 us] short as possible (see d14)
;p16: homospoil/gradient pulse   [1 msec]
;p27: f1 channel - 90 degree pulse at pl18 (same as P1)
;p32: f1 channel - 180 degree shaped pulse (adiabatic)   [20 msec]
;   smoothed chirp (sweepwidth, 20% smoothing, 10000 points) (3ms may also work in complex samples)

;LABEL_ZQS: for zero quantum spoil start experiment with
;   option -D_ZQF (eda: ZGOPTNS)
; filter is recommended for improved lineshape. But can reduce signal in samples with very fast relaxation.

;LABEL_ss : for sustaining singlet state
;   option ZGOPTNS -D_ss (eda: ZGOPTNS)
; filter is to maintain the singlet state. You can use it with cwp or waltz16.
;For the power use at least 3times the chemical shift difference in Hz, centered in the middle of the spin pair resonance.

;LABEL_TOCSY: to turn on TOCSY mixing. Start experiment with
;   option -D_TOCSY (eda: ZGOPTNS)
; TOCSY will recover the complete spin system. This is ideal for comparing the entire spin system against a database for assignment.
; However, TOCSY will lead to signal loss.
; Once assigned, and for monitoring processes, we recommend turning off TOCSY to simplify spectra and increase SNR
; Avoid TOCSY in-vivo to reduce relaxation losses.

;FLAG _W5: for W5 water suppression. Start experiment with
;   option -D_W5 (eda: ZGOPTNS)
; use when water is very broad and problematic.
; can be used separate from, or in combination with, presaturation as required.
; W5 is applied after DREAMTIME so can be used to when one signal is completely under the water if needed (detect on second signal, away from water in this case)

;ns: 8 * n; 32 * n much better
;ds: 8

;for z-only gradients:
;gpz0: ca. 11%
;gpz3: 31%
;gpz11: 34%
;gpz12: 22%
;gpz13: 6%
;gpz14: 21%
;gpz29: 30%

;use gradient files:
;gpnam0: SMSQ10.100
;gpnam3: SMSQ10.100
;gpnam11: SMSQ10.100
;gpnam12: SMSQ10.100
;gpnam13: SMSQ10.100
;gpnam14: SMSQ10.100
;gpnam29: SMSQ10.100

4. References

1 M. Tabatabaei Anaraki, R. Dutta Majumdar, N. Wagner, R. Soong, V. Kovacevic, E. J. Reiner, S. P. Bhavsar, X. Ortiz Almirall, D. Lane, M. J. Simpson, H. Heumann, S. Schmidt, A. J. Simpson, *Anal. Chem.,* 2018, **90**, 7912–7921.
2 M. Piotto, V. Saudek, V. Sklenář, *J. Biomol. NMR,* 1992, **2**, 661–665.
3 B. Lam, A. J. Simpson, *Analyst,* 2008, ***133***, 263–269.
4 S. Mamone, N. Rezaei-ghaleh, F. Opazo, C. Griesinger, S. Glöggler, *Sci. Adv.,* 2020, ***6***, 1–7.
5 V. Govindaraju, K. Young, A. A. Maudsley, *NMR Biomed.,* 2000, **13**, 129–153.
6 J. D. Van Beek, M. Carravetta, G. C. Antonioli, M. H. Levitt, *J. Chem. Phys.,* 2005, **122**, DOI 10.1063/1.1943947.
7 S. Mamone, A. B. Schmidt, N. Schwaderlapp, T. Lange, D. von Elverfeldt, J. Hennig, S. Glöggler, *NMR Biomed.,* 2021, **34**, 1–10.
